# Application of Poisson kriging to the mapping of cholera and dysentery incidence in an endemic area of Bangladesh

**DOI:** 10.1186/1476-072X-5-45

**Published:** 2006-10-13

**Authors:** Mohammad Ali, Pierre Goovaerts, Nushrat Nazia, M Zahirul Haq, Mohammad Yunus, Michael Emch

**Affiliations:** 1 International Vaccine Institute, SNU Research Park, San 4-8 Bongcheon-7 dong, Kwanak-gu, Seoul, Korea; 2 BioMedware, Inc., Ann Arbor, MI, USA; 3 University of Texas at Dallas, USA; 4 ICDDR,B: Centre for Health and Population Research, Dhaka, Bangladesh; 5 University of North Carolina at Chapel Hilll, USA

## Abstract

**Background:**

Disease maps can serve to display incidence rates geographically, to inform on public health provision about the success or failure of interventions, and to make hypothesis or to provide evidences concerning disease etiology. Poisson kriging was recently introduced to filter the noise attached to rates recorded over sparsely populated administrative units. Its benefit over simple population-weighted averages and empirical Bayesian smoothers was demonstrated by simulation studies using county-level cancer mortality rates. This paper presents the first application of Poisson kriging to the spatial interpolation of local disease rates, resulting in continuous maps of disease rate estimates and the associated prediction variance. The methodology is illustrated using cholera and dysentery data collected in a cholera endemic area (Matlab) of Bangladesh.

**Results:**

The spatial analysis was confined to patrilineally-related clusters of households, known as *baris*, located within 9 kilometers from the Matlab hospital to avoid underestimating the risk of disease incidence, since patients far away from the medical facilities are less likely to travel. Semivariogram models reveal a range of autocorrelation of 1.1 km for dysentery and 0.37 km for cholera. This result translates into a cholera risk map that is patchier than the dysentery map that shows a large zone of high incidence in the south-central part of the study area, which is quasi-urban. On both maps, lower risk values are found in the Northern part of the study area, which is also the most distant from the Matlab hospital. The weaker spatial continuity of cholera versus dysentery incidence rates resulted in larger kriging variance across the study area.

**Conclusion:**

The approach presented in this paper enables researchers to incorporate the pattern of spatial dependence of incidence rates into the mapping of risk values and the quantification of the associated uncertainty. Differences in spatial patterns, in particular the range of spatial autocorrelation, reflect differences in the mode of transmission of cholera and dysentery. Our risk maps for cholera and dysentery incidences should help identifying putative factors of increased disease incidence, leading to more effective prevention and remedial actions in endemic areas.

## Background

Disease maps can serve to display incidence rates geographically, to inform on public health provision about the success or failure of interventions, and to make hypothesis or to provide evidences concerning disease etiology [1]. Understanding social/human ecological influence on health may help trigger an effective mechanism to reduce the risk of the disease. However, making a perfect disease risk map is challenging. Recent reviews of disease mapping have been provided by Kulldroff [2], Bithel [3], Diggle [4], Lawson [5], and Lawson and Clark [6]. Most of the proposed methods to create disease maps were tested on data aggregated within large geographical units and applied on non-infectious (cancer-related) diseases. Since ecological process in a large geographical unit would eventually be seen as flat, associating it with the spatial pattern of diseases may yield spurious outcomes [7].

Cholera and other diarrheal diseases remain a problem in many developing countries [8-11]. Endemic cholera has displayed a particular geographical pattern in many nations of the world [11-16]. Although several geographic studies were conducted for studying disease risk, only a few studies were concerned with risk in endemic areas that creates a constant public health problem. Identifying zones of elevated risk in endemic areas and studying socioecological conditions of these zones may provide useful clues for controlling the disease burden and improving the endemic situation. However, assessing risk for diarrheal diseases requires knowledge not only of environment but also of individual's lifestyle and health behavior that may influence the disease risk. Emch [17] illustrated that diarrheal disease risk is a dynamic interaction of biological, socioeconomic, behavioral, cultural and environmental factors over time and space. Heterogeneity in these factors among individuals may influence normal process of the disease phenomena.

Since cholera and other diarrheal diseases are primarily driven by environmental factors [18], and since environmental processes are spatially continuous in nature [19], these disease rates should display some spatial pattern. In other words, rates measured in households that are close geographically should be more similar than the rates recorded over larger separation distances. Geostatistics provides tools to analyze spatially distributed data, capitalizing on the correlation between observations to interpolate the attribute of interest and to delineate areas with high disease risk [1,19,20]. Traditional geostatistical methods are, however, not suited to the analysis of disease rates since they ignore the fact that rate data consist of a denominator and a numerator, which means that observations have varying degree of reliability that should be incorporated into the analysis. Goovaerts [21,22] recently introduced a methodology that is based on Poisson kriging and semivariogram estimators developed by Monestiez et al. [23] for mapping the relative abundance of species in the presence of spatially heterogeneous observation efforts and sparse animal sightings. Simulation studies indicated that this approach outperforms simple population-weighted averages and empirical Bayesian smoothers to estimate underlying risk in cancer mortality maps. The analysis was however confined to rate data aggregated to the level of large geographical entities (i.e. counties) that covered the entire study area, thereby eliminating the need for predicting disease risks at unmonitored locations. The creation of continuous risk maps was addressed in Monestiez et al. [23] with an application to the mapping of the expected number of whale sightings per hour.

This paper presents the first application of Poisson kriging to the spatial interpolation of local disease rates. Cholera and dysentery data were collected at the household level in an endemic area of Bangladesh. Both the disease risk and associated measure of reliability are mapped using geostatistics. These results allow then the mapping of the probability that locally the incidence rate differs from the average rate recorded over the region.

## Methods

### The study area

The study was carried out in Matlab, a field research station of the ICDDR,B (International Centre for Diarrhoeal Disease Research, Bangladesh): Centre for Health and Population Research, Bangladesh. Matlab is situated near the lower Meghna River, one of the main rivers of Bangladesh, and is approximately 50 km southeast of Dhaka (the capital of Bangladesh). The field research station has been involved with cholera studies since 1963. Approximately 200,000 people live in the 184 square kilometers study area. Currently, there are 142 villages; 128 are predominantly Muslim, the rest are predominantly Hindu. The study area is almost entirely rural and most people's occupations are in agriculture or fisheries. The monsoon climate of the study area is characterized by high temperatures, heavy rainfall and marked seasonal variation [24,25].

### Population and disease data

In 1994, a GIS database for the study area was created in order to facilitate spatial analysis for health and population studies. The database includes *baris*, flood-control embankments, rivers, village boundaries, and health facilities. *Baris *are patrilineally-related clusters of households. An average of five households lived in a *bari*. The details on how the geographic database was created can be found elsewhere [26]. This database facilitates the linking of population and disease surveillance data to *bari *locations. The ICDDR,B's Health and Demographic Surveillance Systems (HDSS) databases are maintained at the individual level, thus, the data are aggregated to the *baris*-level for spatial analysis. There were 7,181 points representing *baris *and 207,576 individuals in the study area in 1994. The number of individuals living in a *bari *ranges from 1 to 469, with a median number of 19.

The population data were obtained from the HDSS database. Initiated in 1966, the HDSS maintains records of all vital demographic events [27-29]. The residents in the study area are identified by a registration number given in the surveillance system. To identify the household locations of the individuals, the HDSS maintains a *bari *identification, which is unique and a permanent number in the system.

The disease data were obtained from the Matlab hospital surveillance system, which has in- and out-patient services as well as a laboratory for the identification of pathogens. The Matlab hospital treats approximately 7,000 to 8,000 diarrhea cases per year at no cost to patients. The hospital maintains motorized boats, which function as a free ambulance service for diarrhea patients; access to the hospital is excellent throughout the study area. Stool samples are collected from all patients who are admitted to the hospital who live in the ICDDR,B area. All stool samples are screened for enteric pathogens in the laboratory.

**Figure 1:**
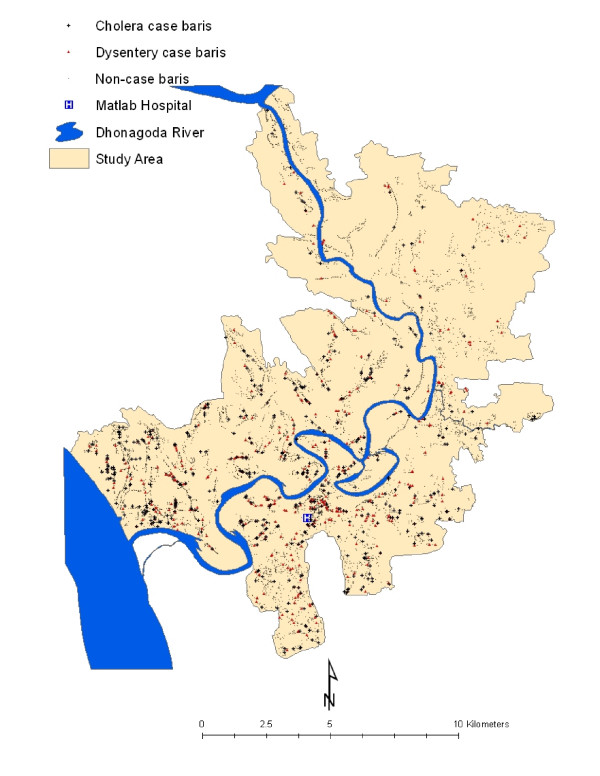
**Spatial distribution of cholera and dysentery in Matlab, 1994**. There were 7181 baris in the study area. In 1994, a total of 706 cholera cases were observed in 455 baris, and the 510 dysentery cases were observed in 421 baris.

We obtained cholera and dysentery data for 1994, the year when the GIS database was created for conducting the study. Note that these two diseases are endemic in that research setting. All cholera cases were laboratory confirmed for *V. cholerea *isolated from subject's stool. A dysentery case was defined as the presence of blood in stool and sought for medical treatment at the Matlab hospital. The spatial distribution of cholera and dysentery cases is shown in Figure 1.

### Disease mapping

Disease mapping requires the interpolation of bari-level rate data to the nodes of a grid covering the study area, in this case a grid with 100 meters spacing. Although spatial interpolation methods, such as kriging, are now fairly common, they cannot be applied directly to disease data since they ignore the population size underlying the computation of rate data. In other words, rates computed from small population sizes (e.g. half of the baris include no more than 19 individuals) tend to be less reliable than rates computed in densely populated areas, and this effect should be incorporated into the estimation algorithm. Poisson kriging allows one to account for population size in the analysis of disease data. The risk of contracting a disease at a given location **u**_*α*_, denoted r(**u**_*α*_), is estimated as a linear combination of K neighboring data:

r^PK(uα)=∑i=1Kλi(uα)z(ui)     (Equation 1)
 MathType@MTEF@5@5@+=feaafiart1ev1aaatCvAUfKttLearuWrP9MDH5MBPbIqV92AaeXatLxBI9gBaebbnrfifHhDYfgasaacH8akY=wiFfYdH8Gipec8Eeeu0xXdbba9frFj0=OqFfea0dXdd9vqai=hGuQ8kuc9pgc9s8qqaq=dirpe0xb9q8qiLsFr0=vr0=vr0dc8meaabaqaciaacaGaaeqabaqabeGadaaakeaacuWGYbGCgaqcamaaBaaaleaacqWGqbaucqWGlbWsaeqaaOGaeiikaGIaeCyDau3aaSbaaSqaaGGaciab=f7aHbqabaGccqGGPaqkcqGH9aqpdaaeWbqaaiab=T7aSnaaBaaaleaacqWGPbqAaeqaaOGaeiikaGIaeCyDau3aaSbaaSqaaiab=f7aHbqabaGccqGGPaqkcqWG6bGEcqGGOaakcqWH1bqDdaWgaaWcbaGaemyAaKgabeaakiabcMcaPaWcbaGaemyAaKMaeyypa0JaeGymaedabaGaem4saSeaniabggHiLdGccaWLjaGaaCzcamaabmaabaGaeeyrauKaeeyCaeNaeeyDauNaeeyyaeMaeeiDaqNaeeyAaKMaee4Ba8MaeeOBa4MaeeiiaaIaeGymaedacaGLOaGaayzkaaaaaa@5B26@

where z(**u**_i_) is the rate observed at location **u**_i_. The kriging weights are found by solving the following system of (K+1) linear equations:

∑j=1Kλj(uα)[CR(ui−uj)+δijm*n(ui)]+μ(uα)=CR(ui−uα)      i=1,...,K∑j=1Kλj(uα)=1
 MathType@MTEF@5@5@+=feaafiart1ev1aaatCvAUfKttLearuWrP9MDH5MBPbIqV92AaeXatLxBI9gBaebbnrfifHhDYfgasaacH8akY=wiFfYdH8Gipec8Eeeu0xXdbba9frFj0=OqFfea0dXdd9vqai=hGuQ8kuc9pgc9s8qqaq=dirpe0xb9q8qiLsFr0=vr0=vr0dc8meaabaqaciaacaGaaeqabaqabeGadaaakqaabeqaamaaqahabaacciGae83UdW2aaSbaaSqaaiabdQgaQbqabaGccqGGOaakcqWH1bqDdaWgaaWcbaGae8xSdegabeaakiabcMcaPmaadmaabaGaem4qam0aaSbaaSqaaiabdkfasbqabaGccqGGOaakcqWH1bqDdaWgaaWcbaGaemyAaKgabeaakiabgkHiTiabhwha1naaBaaaleaacqWGQbGAaeqaaOGaeiykaKIaey4kaSIae8hTdq2aaSbaaSqaaiabdMgaPjabdQgaQbqabaGcdaWcaaqaaiabd2gaTjabcQcaQaqaaiabd6gaUjabcIcaOiabhwha1naaBaaaleaacqWGPbqAaeqaaOGaeiykaKcaaaGaay5waiaaw2faaaWcbaGaemOAaOMaeyypa0JaeGymaedabaGaem4saSeaniabggHiLdGccqGHRaWkcqWF8oqBcqGGOaakcqWH1bqDdaWgaaWcbaGae8xSdegabeaakiabcMcaPiabg2da9iabdoeadnaaBaaaleaacqWGsbGuaeqaaOGaeiikaGIaeCyDau3aaSbaaSqaaiabdMgaPbqabaGccqGHsislcqWH1bqDdaWgaaWcbaGae8xSdegabeaakiabcMcaPiabbccaGiabbccaGiabbccaGiabbccaGiabbccaGiabbccaGiabbMgaPjabg2da9iabbgdaXiabbYcaSiabb6caUiabb6caUiabb6caUiabbYcaSiabdUealbqaamaaqahabaGae83UdW2aaSbaaSqaaiabdQgaQbqabaGccqGGOaakcqWH1bqDdaWgaaWcbaGae8xSdegabeaakiabcMcaPaWcbaGaemOAaOMaeyypa0JaeGymaedabaGaem4saSeaniabggHiLdGccqGH9aqpcqaIXaqmaaaa@8904@

where *δ*_ij _= 1 if **u**_i _= **u**_j _and 0 otherwise, n(**u**_i_) is the population size at **u**_i_, and m* is the population-weighted mean of the set of N rates computed as:

m*=∑α=1Nn(uα)z(uα)∑α=1Nn(uα)     (Equation 3)
 MathType@MTEF@5@5@+=feaafiart1ev1aaatCvAUfKttLearuWrP9MDH5MBPbIqV92AaeXatLxBI9gBaebbnrfifHhDYfgasaacH8akY=wiFfYdH8Gipec8Eeeu0xXdbba9frFj0=OqFfea0dXdd9vqai=hGuQ8kuc9pgc9s8qqaq=dirpe0xb9q8qiLsFr0=vr0=vr0dc8meaabaqaciaacaGaaeqabaqabeGadaaakeaacqWGTbqBcqGGQaGkcqGH9aqpdaWcaaqaamaaqahabaGaemOBa4MaeiikaGIaeCyDau3aaSbaaSqaaGGaciab=f7aHbqabaGccqGGPaqkcqWG6bGEcqGGOaakcqWH1bqDdaWgaaWcbaGae8xSdegabeaakiabcMcaPaWcbaGae8xSdeMaeyypa0JaeGymaedabaGaemOta4eaniabggHiLdaakeaadaaeWbqaaiabd6gaUjabcIcaOiabhwha1naaBaaaleaacqWFXoqyaeqaaOGaeiykaKcaleaacqWFXoqycqGH9aqpcqaIXaqmaeaacqWGobGta0GaeyyeIuoaaaGccaWLjaGaaCzcamaabmaabaGaeeyrauKaeeyCaeNaeeyDauNaeeyyaeMaeeiDaqNaeeyAaKMaee4Ba8MaeeOBa4MaeeiiaaIaeG4mamdacaGLOaGaayzkaaaaaa@608D@

The quantity m*/*n*(**u**_i_) is an error variance term that represents the variability arising from population size and is derived directly under the Poisson model for the counts [21]. The incorporation of this term for a zero distance (i.e. **u**_i _= **u**_j_) leads one to assign smaller kriging weights to rates that are computed from smaller populations and deemed less reliable. The term *μ*(**u**_*α*_) is a Lagrange parameter that results from the minimization of the estimation variance subject to the unbiasedness constraint on the estimator.

To solve the kriging system (Equation 2), one needs to have a model of the spatial covariance of the unknown risk, *C*_*R*_(**h**), or equivalently its semivariogram *γ*_*R*_(**h**) = *C*_*R*_(0)-*C*_*R*_(**h**). The experimental semivariogram of the risk is computed using the following estimator developed by Monestiez et al. [23]:

γ^R(h)=12∑α=1N(h)n(uα)n(uα+h)n(uα)+n(uα+h)∑α=1N(h){n(uα)n(uα+h)n(uα)+n(uα+h)[z(uα)−z(uα+h)]2−m*}     (Equation 4)
 MathType@MTEF@5@5@+=feaafiart1ev1aaatCvAUfKttLearuWrP9MDH5MBPbIqV92AaeXatLxBI9gBaebbnrfifHhDYfgasaacH8akY=wiFfYdH8Gipec8Eeeu0xXdbba9frFj0=OqFfea0dXdd9vqai=hGuQ8kuc9pgc9s8qqaq=dirpe0xb9q8qiLsFr0=vr0=vr0dc8meaabaqaciaacaGaaeqabaqabeGadaaakeaaiiGajugqbiqb=n7aNzaajaGcdaWgaaqcLbuabaqcLboacqWGsbGuaKqzafqabaGaeiikaGIaeCiAaGMaeiykaKIaeyypa0JcdaWcaaqaaKqzafGaeGymaedakeaajugqbiabikdaYOWaaabCaeaadaWcaaqaaKqzafGaemOBa4MaeiikaGIaeCyDauNcdaWgaaWcbaGae8xSdegabeaajugqbiabcMcaPiabd6gaUjabcIcaOiabhwha1PWaaSbaaSqaaiab=f7aHbqabaqcLbuacqGHRaWkcqWHObaAcqGGPaqkaOqaaKqzafGaemOBa4MaeiikaGIaeCyDauNcdaWgaaWcbaGae8xSdegabeaajugqbiabcMcaPiabgUcaRiabd6gaUjabcIcaOiabhwha1PWaaSbaaSqaaiab=f7aHbqabaqcLbuacqGHRaWkcqWHObaAcqGGPaqkaaaaleaacqWFXoqycqGH9aqpcqaIXaqmaeaacqWGobGtcqGGOaakcqWHObaAcqGGPaqka0GaeyyeIuoaaaGcdaaeWbqaamaacmaabaWaaSaaaeaajugqbiabd6gaUjabcIcaOiabhwha1PWaaSbaaSqaaiab=f7aHbqabaqcLbuacqGGPaqkcqWGUbGBcqGGOaakcqWH1bqDkmaaBaaaleaacqWFXoqyaeqaaKqzafGaey4kaSIaeCiAaGMaeiykaKcakeaajugqbiabd6gaUjabcIcaOiabhwha1PWaaSbaaSqaaiab=f7aHbqabaqcLbuacqGGPaqkcqGHRaWkcqWGUbGBcqGGOaakcqWH1bqDkmaaBaaaleaacqWFXoqyaeqaaKqzafGaey4kaSIaeCiAaGMaeiykaKcaaOWaaubiaKqzafqabeqaleaacqaIYaGmaKqzafqaaOWaamWaaKqzafqaaiabdQha6jabcIcaOiabhwha1PWaaSbaaSqaaiab=f7aHbqcLbuabeaacqGGPaqkcqGHsislcqWG6bGEcqGGOaakcqWH1bqDkmaaBaaaleaacqWFXoqyaKqzafqabaGaey4kaSIaeCiAaGMaeiykaKcakiaawUfacaGLDbaaaaqcLbuacqGHsislcqWGTbqBcqGGQaGkaOGaay5Eaiaaw2haaaWcbaGae8xSdeMaeyypa0JaeGymaedabaGaemOta4KaeiikaGccbeGae4hAaGMaeiykaKcaniabggHiLdGccaWLjaGaaCzcamaabmaabaGaeeyrauKaeeyCaeNaeeyDauNaeeyyaeMaeeiDaqNaeeyAaKMaee4Ba8MaeeOBa4MaeeiiaaIaeGinaqdacaGLOaGaayzkaaaaaa@C10B@

where N(**h**) is the number of pairs of baris separated by a vector **h**. The different spatial increments [*z*(**u**_*α*_)-*z*(**u**_*α *_+ **h**)]^2 ^are weighted by a function of their respective population sizes, *n*(**u**_*α*_)*n*(**u**_*α *_+ **h**)/(*n*(**u**_*α*_) + *n*(**u**_*α *_+ **h**)), a term which is inversely proportional to their standard deviation. More importance is thus given to the more reliable data pairs (i.e. smaller standard deviations). A permissible model, *γ *_*R*_(**h**), is then fitted to the experimental semivariogram in order to derive the semivariogram, or covariance value, for any possible distance h. In this paper, the modeling was conducted using the weighted least-square regression procedure implemented in the public-domain software **poisson_kriging.exe **[21].

A probabilistic model of the uncertainty about the risk of incidence of the disease at a given location **u**_*α*_, denoted r(**u**_*α*_), is provided by the random variable *R*(**u**_*α*_). This variable is fully characterized by its conditional cumulative distribution function (ccdf) defined as:

F(uα;r|(K))=Pr⁡ob{R(uα)≤r|(K)}=G(r−r^PK(uα)σPK2(uα))     (Equation 5)
 MathType@MTEF@5@5@+=feaafiart1ev1aaatCvAUfKttLearuWrP9MDH5MBPbIqV92AaeXatLxBI9gBaebbnrfifHhDYfgasaacH8akY=wiFfYdH8Gipec8Eeeu0xXdbba9frFj0=OqFfea0dXdd9vqai=hGuQ8kuc9pgc9s8qqaq=dirpe0xb9q8qiLsFr0=vr0=vr0dc8meaabaqaciaacaGaaeqabaqabeGadaaakeaacqWGgbGrcqGGOaakcqWH1bqDdaWgaaWcbaacciGae8xSdegakeqaaiabcUda7iabdkhaYjabcYha8jabcIcaOiabdUealjabcMcaPiabcMcaPiabg2da9iGbccfaqjabckhaYjabb+gaVjabbkgaInaacmaabaGaemOuaiLaeiikaGIaeCyDau3cdaWgaaqaaiab=f7aHbqabaGccqGGPaqkcqGHKjYOcqWGYbGCcqGG8baFcqGGOaakcqWGlbWscqGGPaqkaiaawUhacaGL9baacqGH9aqpcqWGhbWrdaqadaqaamaalaaabaGaemOCaiNaeyOeI0IafmOCaiNbaKaadaWgaaWcbaGaemiuaaLaem4saSeabeaakiabcIcaOiabhwha1naaBaaaleaacqWFXoqyaOqabaGaeiykaKcabaWaaOaaaeaacqWFdpWCdaqhaaWcbaGaemiuaaLaem4saSeabaGaeGOmaidaaOGaeiikaGIaeCyDau3aaSbaaSqaaiab=f7aHbGcbeaacqGGPaqkaSqabaaaaaGccaGLOaGaayzkaaGaaCzcaiaaxMaadaqadaqaaiabbweafjabbghaXjabbwha1jabbggaHjabbsha0jabbMgaPjabb+gaVjabb6gaUjabbccaGiabiwda1aGaayjkaiaawMcaaaaa@7839@

where *G*(.) is the cumulative distribution function of the standard normal random variable. The notation "|(*K*)" expresses conditioning to the local information, say, *K *neighboring observed rates. The ccdf is modeled as a Gaussian distribution [22] with the mean and variance corresponding to the Poisson kriging estimate (Equation 1) and the kriging variance that is computed as:

σPK2(uα)=CR(0)−∑i=1Kλi(uα)CR(ui−uα)−μ(uα)     (Equation 6)
 MathType@MTEF@5@5@+=feaafiart1ev1aaatCvAUfKttLearuWrP9MDH5MBPbIqV92AaeXatLxBI9gBaebbnrfifHhDYfgasaacH8akY=wiFfYdH8Gipec8Eeeu0xXdbba9frFj0=OqFfea0dXdd9vqai=hGuQ8kuc9pgc9s8qqaq=dirpe0xb9q8qiLsFr0=vr0=vr0dc8meaabaqaciaacaGaaeqabaqabeGadaaakeaaiiGacqWFdpWCdaqhaaWcbaGaemiuaaLaem4saSeabaGaeGOmaidaaOGaeiikaGIaeCyDau3aaSbaaSqaaiab=f7aHbqabaGccqGGPaqkcqGH9aqpcqWGdbWqdaWgaaWcbaGaemOuaifabeaakiabcIcaOiabicdaWiabcMcaPiabgkHiTmaaqahabaGae83UdW2aaSbaaSqaaiabdMgaPbqabaGccqGGOaakcqWH1bqDdaWgaaWcbaGae8xSdegabeaakiabcMcaPiabdoeadnaaBaaaleaacqWGsbGuaeqaaOGaeiikaGIaeCyDau3aaSbaaSqaaiabdMgaPbqabaGccqGHsislcqWH1bqDdaWgaaWcbaGae8xSdegabeaakiabcMcaPaWcbaGaemyAaKMaeyypa0JaeGymaedabaGaem4saSeaniabggHiLdGccqGHsislcqWF8oqBcqGGOaakcqWH1bqDdaWgaaWcbaGae8xSdegabeaakiabcMcaPiaaxMaacaWLjaWaaeWaaeaacqqGfbqrcqqGXbqCcqqG1bqDcqqGHbqycqqG0baDcqqGPbqAcqqGVbWBcqqGUbGBcqqGGaaicqaI2aGnaiaawIcacaGLPaaaaaa@6F23@

The ccdf (Equation (5)) allows the computation and mapping of the probability that the local incidence rate exceeds any particular threshold. In absence of regulatory threshold in the present case study, this function is here used to assess whether the local incidence rate at **u**_*α *_is significantly different from the regional rate m* (Equation 3). The *p*-value for the test is calculated as:

p(uα)=2[1−G(|m*−r^PK(uα)σPK2(uα)|)]     (Equation 7)
 MathType@MTEF@5@5@+=feaafiart1ev1aaatCvAUfKttLearuWrP9MDH5MBPbIqV92AaeXatLxBI9gBaebbnrfifHhDYfgasaacH8akY=wiFfYdH8Gipec8Eeeu0xXdbba9frFj0=OqFfea0dXdd9vqai=hGuQ8kuc9pgc9s8qqaq=dirpe0xb9q8qiLsFr0=vr0=vr0dc8meaabaqaciaacaGaaeqabaqabeGadaaakeaacqWGWbaCcqGGOaakcqWH1bqDdaWgaaWcbaacciGae8xSdegakeqaaiabcMcaPiabg2da9iabikdaYmaadmaabaGaeGymaeJaeyOeI0Iaem4raC0aaeWaaeaadaabdaqaamaalaaabaGaemyBa0MaeiOkaOIaeyOeI0IafmOCaiNbaKaadaWgaaWcbaGaemiuaaLaem4saSeabeaakiabcIcaOiabhwha1naaBaaaleaacqWFXoqyaOqabaGaeiykaKcabaWaaOaaaeaacqWFdpWCdaqhaaWcbaGaemiuaaLaem4saSeabaGaeGOmaidaaOGaeiikaGIaeCyDau3aaSbaaSqaaiab=f7aHbGcbeaacqGGPaqkaSqabaaaaaGccaGLhWUaayjcSdaacaGLOaGaayzkaaaacaGLBbGaayzxaaGaaCzcaiaaxMaadaqadaqaaiabbweafjabbghaXjabbwha1jabbggaHjabbsha0jabbMgaPjabb+gaVjabb6gaUjabbccaGiabiEda3aGaayjkaiaawMcaaaaa@6474@

Because the test is repeated over a large number of grid node **u**_*α*_, some locations are expected to be tested significant even if there is no significant differences present (i.e. multiple testing). Rather than using the very conservative Bonferroni correction, *p*-values were here simply used to rank locations according to their likelihood of having much smaller or larger incidence rates without any formal classification as significant locations or not.

## Results and discussion

**Figure 2:**
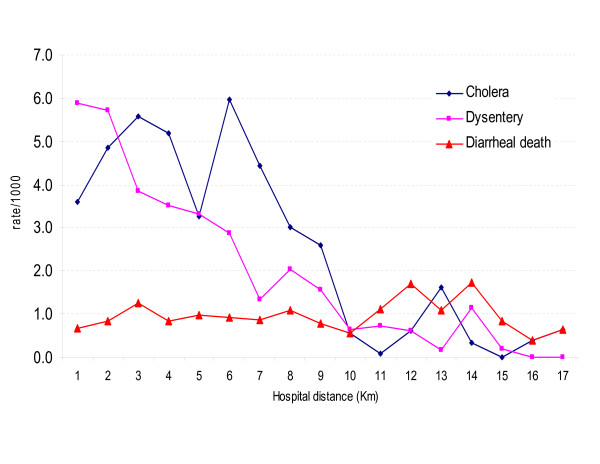
**Hospital admittance rate for cholera and dysentery, and death due to diarrheal death (watery) in Matlab as a function of the distance to the Hospital, 1994**. The morbidity data were obtained from hospital-based disease surveillance, while the mortality data were obtained from community based demographic surveillance. All the rates are expressed as number of cases per 1,000 habitants.

Figure 2 shows admission rates for both cholera and dysentery patients, as well as death due to acute watery diarrhea, as a function of the distance to the Matlab hospital. Mortality data were collected through routine demographic surveillance programs that ensure completeness of the data for the entire study area. Unlike mortality rates due to acute watery diarrhea, the two incidence rates decline to a near zero level when the bari is more than 9 km away from the hospital. This strong contrast between mortality and disease incidence rates highlights the impact of proximity to medical facilities on hospital admittance; hence the risk of bias in mapping disease rates in remote areas. The following analysis has thus been restricted to data collected in bari within a radius of 9 kilometers from the hospital.

**Figure 3:**
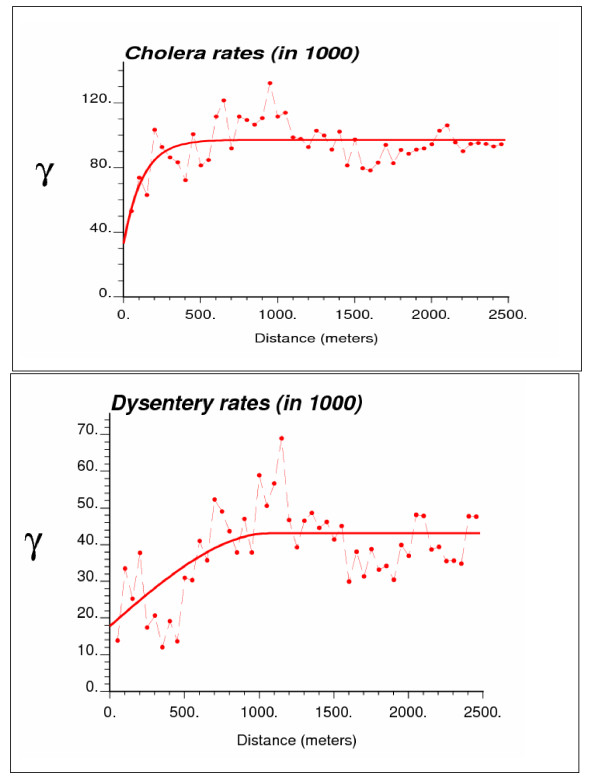
Omnidirectional semivariograms of cholera and dysentery incidence rates, with the model fitted.

Experimental risk semivariograms were computed from the 6,017 bari-level incidence rates using the population-weighted estimator (Equation 4). Since no systematic difference was observed between directional semivariograms, the spatial variability was considered isotropic and only the omnidirectional semivariograms are displayed in Figure 3. For both diseases, the semivariogram was estimated using 50 lags of 50 meters. The modeling was performed using weighted least-square regression where the weights account for both the number of data pairs and the semivariogram value (i.e. weighting option #2, w(**h**_l_) = N(hl)
 MathType@MTEF@5@5@+=feaafiart1ev1aaatCvAUfKttLearuWrP9MDH5MBPbIqV92AaeXatLxBI9gBaebbnrfifHhDYfgasaacH8akY=wiFfYdH8Gipec8Eeeu0xXdbba9frFj0=OqFfea0dXdd9vqai=hGuQ8kuc9pgc9s8qqaq=dirpe0xb9q8qiLsFr0=vr0=vr0dc8meaabaqaciaacaGaaeqabaqabeGadaaakeaadaGcaaqaaiabd6eaojabcIcaOiabhIgaOnaaBaaaleaacqWGSbaBaeqaaOGaeiykaKcaleqaaaaa@3292@/*γ*(**h**_l_), in the program **poisson_kriging.exe**).

The cholera semivariogram model is exponential with a practical range of 370 meters, while a spherical model with a range of 1,100 meters was fitted for dysentery. The larger range of autocorrelation for the second model indicates a better spatial structure of dysentery incidence rates, which can be explained by the differences in the mode of transmission for the two diseases. Cholera is usually transmitted to a person through oral-fecal-oral process, while dysentery can be spread through hand-to-hand contacts. In the past there have been a number of interventions carried out in the study area to reduce the risk of cholera, while dysentery received much less attention. Therefore, local differences in the socioeconomic condition and health behavior among neighboring households due to health interventions may explain the shorter range of spatial autocorrelation displayed by cholera incidence rates relatively to dysentery.

**Figure 4:**
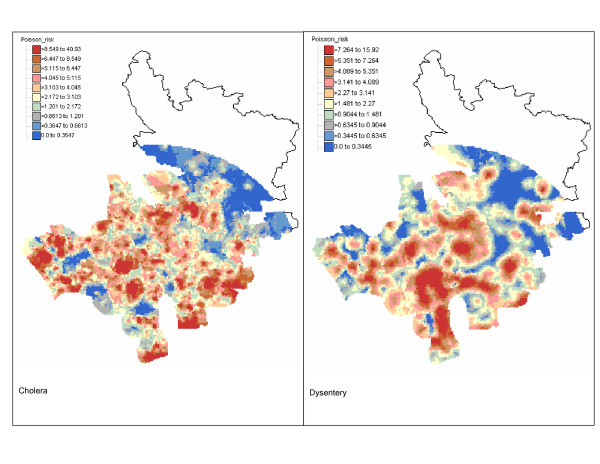
**Maps of cholera and dysentery incidence risk in Matlab 1994 estimated by Poisson kriging**. The class boundaries of the color legend correspond to the deciles of the histogram of risk estimates.

The risk of incidence of both diseases was mapped over a grid with a 100-meter spacing. All 13,689 grid nodes are within a 9 km distance from the Matlab hospital. At each node, up to a maximum of 64 closest observations within a radius of 5 km were used for Poisson kriging. Figure 4 shows the maps of cholera and dysentery risk. In both cases, the boundaries of the color classes correspond to the deciles of the histogram of risk estimates. Table 1 indicates that, on average over all grid nodes, the kriged risks are slightly smaller than the observed rates. Yet, the extreme high, and likely unstable, incidence rates (i.e. 500 per 1,000 habitants for cholera and 250 per 1,000 habitants for dysentery) are not reproduced by the risk maps that display a smaller range of variation. The cholera risk map is patchier than the dysentery map that shows a large zone of high incidence in the south-central part of the study area, which is a quasi-urban zone. This could be due to household level differences in the socioeconomic conditions and health behavior described above. On both maps, lower risks are found in the Northern part of the study area, which is also the most distant from the Matlab hospital. This can partially be explained by the inverse relationship between hospital admittance and distance to the medical facility which is illustrated in Figure 2.

**Table 1:** Descriptive statistics of the distribution of incidence rates and kriged risks for cholera and dysentery in Matlab, 1994.

**Statistics**	**Cholera**		**Dysentery**	
	
	**Kriged risk**	**Observed rate**	**Kriged risk**	**Observed rate**
N	13689	6017	13689	6017
Mean	3.7238	3.8512	2.6841	2.7732
Std. Deviation	3.5083	20.134	2.4847	14.776
Minimum	0.00	0.00	0.00	0.00
Maximum	40.93	500.00	15.92	250

Units are number of cases per 1,000 habitants.

**Figure 5:**
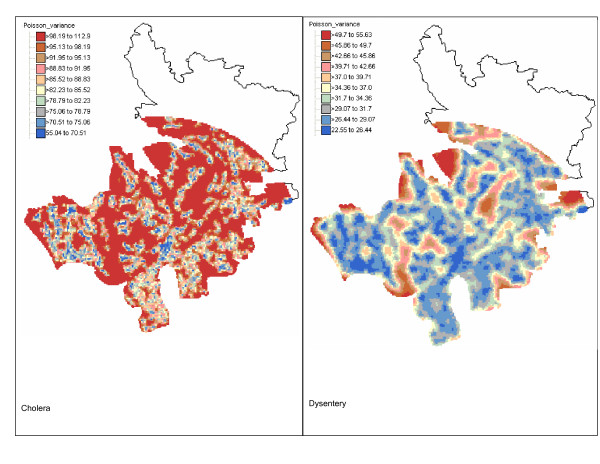
**Maps of Poisson kriging variance for cholera and dysentery incidence risk in Matlab 1994**. The class boundaries of the color legend correspond to the deciles of the histogram of kriging variances.

The kriging variance maps (Figure 5) capture both the spatial distribution of the population across the study area and the spatial pattern of the incidence rates. The greater magnitude of the kriging variance computed for cholera is caused by the shorter range of autocorrelation of its semivariogram model: 0.37 km versus 1.1 km for dysentery. The larger kriging variance alongside the border of the study area highlights lower density of the population.

**Figure 6:**
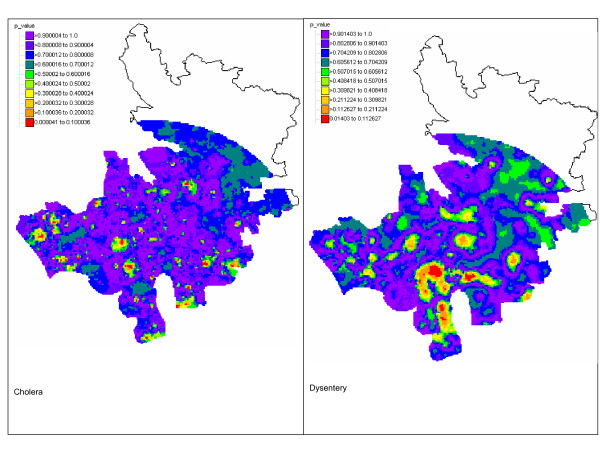
**Maps of the p-values for the test of uniformity of local incidence rates for cholera and dysentery in Matlab 1994**. At each location, the population-weighted mean of all bari rates is compared to the probability distribution of the local incidence rate which is fully characterized by the Poisson kriging estimate and variance. The lower the p-value, the more likely the local rate is significantly smaller or higher than the regional rate.

The kriging variance maps were combined with the risk maps to assess whether the local disease rate is significantly different from the regional rate. For both diseases, only a few grid nodes have a p-value (Equation 6) smaller than 0.05; see Figure 6. Because of multiple testing, these positive results are likely caused by chance and we can safely assume that no local incidence rate departs significantly from the regional rates. Yet, the maps in Figure 6 can be used to rank areas according to the likelihood that the local rate is much smaller or larger than the regional rate. This information should prove useful to guide future control activities.

## Conclusion

In Matlab, disease maps have never been used to isolate areas of high cholera or dysentery incidence and to guide for control activities. The health authority of the area could however greatly benefit from the availability of such maps for their disease control programs. Spatial interpolation of incidence rates can be performed using a variety of simple techniques, including inverse square distance methods, spline or moving average. The main limitation of these deterministic methods is that they fail to provide a measure of the reliability of the prediction. Kriging is increasingly preferred because it accounts for spatial patterns and data configuration (i.e. clustering of observations) in the prediction, while providing both an estimate and a measure of the error variance. The kriging variance can be combined with the kriging estimate to compute confidence intervals or the probability of exceeding specific thresholds, under the assumption of Gaussianity.

Interpolation of bari-level data cannot be performed using conventional geostatistical tools that ignore the population size underlying the computation of incidence rates. Because the number of individuals living in bari greatly fluctuates across the study area, local incidence rates have various degrees of reliability that need to be accounted for in the variography and spatial interpolation. Poisson kriging, which was introduced in the health science literature for filtering the noise in cancer mortality maps, is here used to create continuous maps of incidence risks from punctual bari-level data.

Unlike traditional semivariogram estimators, the version used in this paper directly accounts for population size in the computation of the semivariograms, attenuating the influence of less reliable rates recorded in sparsely populated areas. Cholera and dysentery display semivariograms with very different ranges of autocorrelation. The much larger range (1.1 km versus 0.370 km) observed for dysentery indicates that its incidence rates vary more continuously in space than cholera rates across the study area.

The maps of disease risk illustrated the better spatial continuity of rates recorded for dysentery relatively to cholera. Combined with the different location of high-incidence areas, these contrasted spatial patterns suggest that different mechanisms are responsible for the occurrence of these two diseases. Within the field of spatial epidemiology, knowledge about host, agent and environment is needed to get insight into the etiology of the disease and to develop and promote disease control measures [30]. This means that spatial epidemiological studies have to characterize spatial patterns of disease risk. Spatial variations of disease risk results in clustering of disease, and that may reflect the existence of specific subpopulations that are under higher or lower exposure to the disease. Our risk maps for cholera and dysentery may be useful for identifying putative factors of increased disease risk and for helping health officials take remedial actions.

The high incidence rates for dysentery in the urban part of the study area can be attributed to food habit pattern given that habitants of the study area use their hands to eat. Those who live close to urban areas have easy access to restaurants which tend to ignore basic hygienic rules. Thus, eating meals that are not served hygienically at those places increases the risk of acquiring dysentery. Our dysentery risk map suggests the need of interventions for combating the threats of dysentery in the urban part of the study area. On the other hand, the isolated clusters of high cholera incidence may be attributed to the poor health behavior (e.g. hands are not washed systematically after bathroom break and before eating, drinking water is not boiled or filtered, cooking utensils are washed with surface water, etc.) of the community. We can not also ignore the possibility of environmental niche of endemic cholera in those areas. Further investigations focusing on the areas of high incidence risk for cholera could shed some light on the factors that cause these local spikes in cholera incidence.
